# Value of Vaccination

**Published:** 1873-08

**Authors:** 


### VALUE OF VACCINATION.

BEFORE Jenner's time the deaths from small pox in Copenhagen averaged 460 a year. When vaccination became general only nine died annually from small pox. For the same reason the deaths in London went from 7,500 annually to 384. Of 29,000 soldiers vaccinated in the Prussian army not one had the small pox, and only one varioloid—that is, real small pox, modified by vaccination—real small pox attacking a vaccinated person. And yet, when vaccination decreased the deaths from small pox from 7,500 to less than 400 a year, there are persons who assert that vaccination is injurious to national health, by introducing the poison of disease into the vaccinated person from the one from which the vaccine matter was taken ; but that this is the merest conjectural assumption possible is proven from the fact that, in all standard medical works printed since the practice of vaccination, not one single case is found to show that a body was infected by disease from vaccine matter. On the other hand, it is an accepted fact that a person who has had small pox has a more vigorous constitution ever after, and is less liable to any of the ordinary forms of disease; hence it is reasonable to suppose that vaccination has a similar effect, although in less degree.

In the attempt lately made in London to compel vaccination at the expense of the Government, many became riotous in their opposition.

There are 30,000 births in New York city every year, and the people should be educated to the importance and value of vaccination. All children should be vaccinated within a month of birth, and, for greater safety, this should be repeated at fourteen.

